# Overexpression of* CaAPX* Induces Orchestrated Reactive Oxygen Scavenging and Enhances Cold and Heat Tolerances in Tobacco

**DOI:** 10.1155/2017/4049534

**Published:** 2017-03-13

**Authors:** Jiangying Wang, Bin Wu, Hengfu Yin, Zhengqi Fan, Xinlei Li, Sui Ni, Libo He, Jiyuan Li

**Affiliations:** ^1^Research Institute of Subtropical Forestry, Chinese Academy of Forestry, Zhejiang Provincial Key Laboratory of Tree Breeding, Hangzhou, Zhejiang 311400, China; ^2^Lianyungang Academy of Agricultural Sciences, Flower Research Center, Lianyungang, Jiangsu 222000, China; ^3^School of Marine Sciences, Ningbo University, Ningbo, Zhejiang 315211, China; ^4^College of Horticulture and Landscape Architecture, Hunan Agricultural University, Changsha, Hunan 410128, China

## Abstract

Ascorbate peroxidase (APX) acts indispensably in synthesizing L-ascorbate (AsA) which is pivotal to plant stress tolerance by detoxifying reactive oxygen species (ROS). Enhanced activity of APX has been shown to be a key step for genetic engineering of improving plant tolerance. However it needs a deeper understanding on the maintenance of cellular ROS homeostasis in response to stress. In this study, we identified and characterized an* APX* (*CaAPX*) gene from* Camellia azalea*. Quantitative real-time PCR (qRT-PCR) analysis showed that* CaAPX* was expressed in all tissues and peaked in immature green fruits; the expression levels were significantly upregulated upon cold and hot stresses. Transgenic plants displayed marked enhancements of tolerance under both cold and heat treatments, and plant growth was correlated with* CaAPX* expression levels. Furthermore, we monitored the activities of several ROS-scavenging enzymes including* Cu/Zn-SOD*,* CAT*,* DHAR*, and* MDHAR*, and we showed that stress tolerance was synchronized with elevated activities of ROS-scavenging. Moreover, gene expression analysis of ROS-scavenging enzymes revealed a role of* CaAPX* to orchestrate ROS signaling in response to temperature stresses. Overall, this study presents a comprehensive characterization of cellular response related to* CaAPX* expression and provides insights to breed crops with high temperature tolerances.

## 1. Introduction

Temperature stress, such as heat, cold, or freezing, causes an increase in reactive oxygen species (ROS) levels and induces the oxidative stress in plant cells [1]. The elevation of reactive ROS levels can cause deleterious damage to organelle function and cellular metabolism or leads to programed cell death [2]. To quickly remove excessive ROS, plants evolved a complex system containing nonenzymatic antioxidants, such as ascorbate (AsA), glutathione (GSH), flavonoids and carotenoids, and ROS-scanvenging enzymes including superoxide dismutase (SOD), peroxidase (POD), catalase (CAT), and enzymes in the water-water cycle [3]. The antioxidants and enzymes need to act synergistically to keep the homeostasis of cellular redox state. For example, the water-water cycle, also known as Halliwell-Asada or AsA-GSH cycle, includes the activity of APX, DHAR, GR, and MDHAR and was a pathway involved in scavenging superoxide radicals and H_2_O_2_ [4].

There is an effective regulatory network containing multiple pathways in plant cell to deal with cellular ROS efficiently. The regulation of antioxidants and enzymatic activities was involved in a signaling transduction leading to changes of gene expression levels. Recently the ROS were revealed as central signaling components to orchestrate the multiple scavenging genes expressions under various stressed conditions [5, 6]. Upon temperature stress, the cellular ROS levels were upregulated through the “temperature sensor” which amplified the signal to activate the ROS-scavenging enzymes [2, 7]. Heat shock proteins (HSPs) and heat shock transcription factors (HSFs) were thought to be responsible for temperature stress sensing [8, 9]. On the other hand, the NADPH oxidases (respiratory burst oxidase homologous, RBOHs) played a key role in generating ROS upon stress stimuli, which were involved in different signaling pathways [10, 11]. In Arabidopsis, functional analyses of HSF3 and HSF21 revealed that ROS-scavenging enzymes APX1 and APX2 were imminent targets which were required to protect against heat induced oxidative damage [12, 13]. In addition, APX could act interactively with RBOHD to generate systematic signals of ROS tolerance [10, 13].

Ascorbate peroxidase (APX) was found to be a key enzyme in the ascorbate-glutathione pathway to scavenge cellular H_2_O_2_ produced in various stressful conditions [5, 14–19]. When APX reduces hydrogen peroxide to water by utilizing AsA as an electron donor, monodehydroascorbate (MDA) accumulates. Meanwhile MDA is somewhat unstable and likely to be rereduced disproportionately into AsA and dehydroascorbate (DHA), and both MDA and DHA can be reduced back to AsA through the activity of reductases [20–22]. Many studies over the years have demonstrated that APX played a specific role in improving plant's tolerance response to certain abiotic stresses [19, 23–25]. Enhanced expression of* APX* in transgenic plants could intensify their resistance to multiple environmental stresses through eliminating H_2_O_2_ [19, 23–27]. For example,* APX* overexpression improved chilling tolerances in rice and sweet potato [28, 29]; the transgenic potato plants with APX expression were more tolerant to high temperature stress [30, 31]. In addition, after exposure to some environmental stresses, the* APX* knockdown plants exhibited much more severe cellular injuries [32–34].

Although the genetic manipulation of ROS has been practiced in many plant species, our understanding of ROS removing and signaling remains to be expanded to better improve the plant performance by the genetic manipulation approach. Here, in order to investigate the potential improvements of temperature stresses in* Camellia* species, we cloned* APX* gene from* Camellia azalea* and investigated its expression pattern. We showed that* CaAPX* was expressed in all examined* Camellia* tissues and was quickly induced by the temperature stresses. Functional analysis in transgenic tobacco proved that overexpression of* CaAPX* enhanced plant performances in both cold and heat conditions. Furthermore, we demonstrated that overexpression of* CaAPX* altered the cellular content of AsA, MDA, and H_2_O_2_ concentrations and led to lower electrolyte leakage rate. Enzymatic activity analysis of APX, SOD, POD, and CAT and gene expression analysis of tobacco endogenous Cu/Zn-SOD, CAT, DHAR, and MDHAR genes revealed that CaAPX may have a central role in synchronizing cellular ROS-scavenging network. This work may provide useful information for engineering plants of high resistance to oxidative damage under temperature stresses.

## 2. Materials and Methods

### 2.1. Plant Materials and Treatments

*C. azalea* materials selected for present study including four-year-old grafted seedlings and two-year-old cutting seedlings were collected from a greenhouse of the Research Institute of Subtropical Forestry located in Fuyang (119°57′ E, 30°04′ N; Fuyang city, Zhejiang, China), growing under natural light condition with regular irrigation. And subculture aseptic tobacco seedlings with six true leaves (*Nicotiana tabacum* L., cv. SR1) were grown in a growth chamber at 25°C and 1200 Lux light (16 h light/8 h dark). For treatments, plants were exposed to 4°C low and 38°C high temperatures for 0, 2, 4, 8, and 12 h, respectively, in the growth chamber at 25°C and 1200 Lux light (16 h day/8 h night). The samples treated for 0 h were set as controls. Plant tissues of* C. azalea* seedlings and the forth leaves of treatment tobacco plants collected were immediately put into liquid nitrogen and stored at –80°C for later use.

### 2.2. Cloning of* CaAPX* Gene

Total RNA was extracted using the plant total RNA isolation Kit (Aidlab Biotech, China) for reverse transcription polymerase chain reaction (RT-PCR) (Takara, China). To isolate the* APX* gene, the 3′,5′-RACE amplification Kit (Takara, China) was used to construct libraries from* C. azalea* young leaves. Two amplified fragments (gene-specific primers were listed in Table S1 (see Table S1 in Supplementary Material available online at https://doi.org/10.1155/2017/4049534)) were cloned into pGEM-Teasy vector (Promega, USA) for sequencing, and full-length cDNA sequence was assembled. The cDNA sequence was designated as* CaAPX* and submitted to GenBank (accession number KP635267) (https://www.ncbi.nlm.nih.gov/genbank/).

### 2.3. Quantitative Real-Time PCR

DNA traces of the total RNA were removed by DNase I (RNase-free) (TransGen Biotech, China). The cDNAs from different organs of* C. azalea* cutting seedlings on, respectively, different temperature stress levels were obtained by employing the PrimeScript™ RT reagent Kit (Perfect Real Time) (Takara, China). The* 18S RNA* gene of Camellias was used as an internal control to normalize the amount of mRNA present in each sample. And all samples were amplified in four independent replicates by an Applied Biosystems 7300 Fast Real-Time PCR system using SYBR-*Premix Ex* Taq™ II (Tli RNadeH Plus) (Takara, China) for fluorescence detection and the expression data was analyzed using 2^−ΔΔCt^ method.

### 2.4. Construction of the Plant Expression Vector and Transformation of Tobacco

Full-length cDNA sequence of* CaAPX* gene was obtained by amplification of primers containing* Xba*I and* BamH*I restriction sites (Table S1). The PCR product was integrated into pGEM-Teasy vector (Promega) and then cloned into vector pBI121 by* Xba*I and* BamH*I double digestion. Tobacco transformation through* Agrobacterium*-*tumefaciens-*mediated was performed as the leaf disk method described by Horsch et al. [35], and transgenic plants were screened by 50 mg/L kanamycin.

### 2.5. Southern Blot Analysis

Genomic DNAs (about 25 *μ*g) were obtained from three independent T1 transgenic plant lines and double digested with restriction endonuclease* EcoR*I and* BamH*I (TaKaRa) at 37°C for 20 h and electrophoretically separated on a 0.8% agarose gel and transferred to positively charged nylon membrane.* Hind*III-digested lambda DNA with digoxigenin labeling (Cat. 11218590910, Roche) was employed as marker. The DNA was fixed on the membrane by baking at 120°C for 30 min. According to the protocol of DIG-High Prime DNA Labeling and Detection Starter Kit (Roche, USA), all procedures including the preparation of probe, prehybridization, hybridization, and immunological detection were carried out in proper sequence.

### 2.6. Cold and Heat Stress Tolerance Assays

Three independent T1 transgenic plant lines and WT tobacco plants were incubated on tissue culture medium under a 16 h light/8 h dark cycle at 25°C for 30 d and then transferred into two different growth chambers separately at 4°C and 38°C, 1200 Lux light (16 h day/8 h dark), for 12 h treatment. The treated plants were photographed every two hours, and the forth functional leaves taken at the same time were exploited to conduct physiological and gene expression experiments.

### 2.7. Determination of APX, SOD, POD, and CAT Activity and AsA Concentration

APX activity was determined according to the method of Nakano and Asada [36]. 0.4 g ground frozen fresh samples were homogenized in 4 mL of 50 mM extraction PBS buffer (pH 7.8) containing 0.2 mM EDTA and 2 mM AsA. The homogenate was transferred into a tube and centrifuged at 12,000 ×g for 20 min (4°C) and the supernatant was collected for APXase activity analysis. AsA content was determined according to the method of Arakawa et al. [37]. The crude extracts homogenized through grinding 0.3 g fresh samples macerated with liquid nitrogen in presence of 3 mL 5% (w/v) TCA into a fine powder were centrifuged at 4,000 ×g for 10 min at room temperature and the supernatant was collected for AsA content analysis.

0.2 g ground frozen fresh samples were homogenized in 10 mL precooling PBS buffer (pH 7.8), followed by being centrifuged at 12,000 ×g for 15 min (4°C), and the supernatant was extracted for enzyme activity analysis. SOD, POD, and CAT activities measurement was performed as described by Lee [38].

### 2.8. Biochemical and Physiological Analyses

MDA content was determined by the thiobarbituric acid- (TBA-) based colorimetric method as described by Heath and Packer [39]. H_2_O_2_ content was assayed by modified method according to Shi et al. [40]. 0.5 g fine grinding fresh samples were homogenized in an ice bath with 0.1% (w/v) TCA, and the homogenate was centrifuged at 12,000 ×g for 15 min. Then, 1 mL supernatant was mixed together with 1 mL potassium phosphate buffer (100 mM, pH 7.0) and 2 mL KI (1 M), and the absorbance was measured at 390 nm using a standard curve plotted with known concentration of H_2_O_2_. Relative electric conductivity was measured referring to the method of Zhang [41]. 0.5 g fresh leaf discs (0.5 × 0.5 cm) were cut into several conical flasks containing 25 mL distilled water and vacuumed for 8 min, 6 conical flasks totally, and the conical flasks were, respectively, treated under different temperatures lasting 20 min to determine the initial electric conductivity (*R*) using Conductivity Meter (HI-9033). Then, the whole sample mixtures were heated with boiling water for 15 min to kill the cells and final electric conductivity (*R*_0_) was determined after 2 h later, with electric conductivity of plant leaf dicks at 25°C as a control group (CK). Relative electric conductivity (REC) was evaluated as REC  (%) = [(*R* − CK)/(*R*_0_ − CK)] × 100%.

For photosynthetic analysis, a Chlorophyll Fluorescence Analyzer (Imaging-PAM-Maxi, Walz, Germany) was used to determine all of the parameters according to the manufacturer's instructions.

## 3. Results

### 3.1. Molecular Cloning and Characterization of* CaAPX*

A fragment (566-bp) was obtained by amplification of primers (Table S1) designed based on conserved regions, and sequence analysis showed that it was homologous to* APX* genes (not shown). The full-length sequence of* CaAPX* (GenBank Accession ID: KP635267) was retrieved by 3′ and 5′-RACE amplification (primers listed in Table S1).* CaAPX* cDNA consisted of 1,017-bp nucleotides and encoded an open reading frame of 250 amino acid residues (Figure S1).

**Figure 1:**
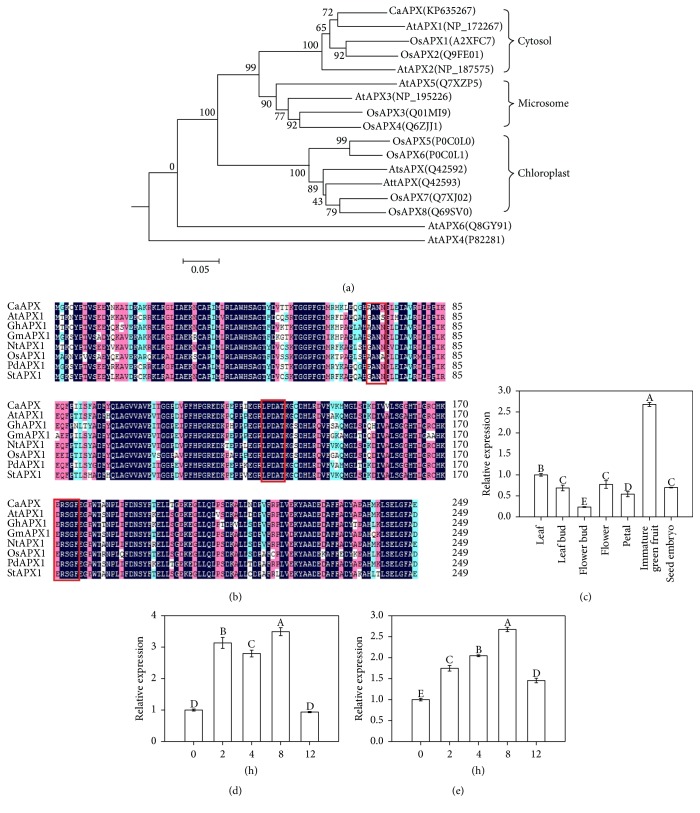
Sequence analysis and gene expression profiles of CaAPX. (a) Phylogenetic tree analysis of CaAPX and related proteins. (b) Alignment of amino acid sequences. Ca,* C. azalea* (KP635267); At,* Arabidopsis thaliana* (NP_172267); Gh,* Gossypium hirsutum* (ABR18607); Gm,* Glycine max* (NP_001237785); Nt,* Nicotiana tomentosiformis* (XP_009597491); Os,* Oryza sativa* (japonica cultivar group) (P93404); Pd,* Phoenix dactylifera* (XP_008783664); St,* Solanum tuberosum* (NP_001275066). Red boxes indicate the highly conserved APX active-site signature. (c) Relative expression of* CaAPX* in different tissues of C. azalea. (d) Relative expression of CaAPX in leaves of* C. azalea* during different times under 4°C cold stress. (e) Relative expression of* CaAPX* in leaves of* C. azalea* during different times under 38°C hot stress. The data are the means of four replicates and were compared by Duncan's multiple range tests.

The deduced amino acid sequence of* CaAPX* was aligned with homologous sequences from various species, including* Arabidopsis thaliana*,* Gossypium hirsutum*,* Glycine max*,* Nicotiana* tomentosiformis,* Oryza sativa*,* Phoenix dactylifera*, and* Solanum tuberosum*, using BLAST (https://blast.ncbi.nlm.nih.gov/Blast.cgi). The predicted structure of CaAPX dominantly contained alpha-helix and random coil, in which a POD domain containing the highly conserved amino acid sequences, namely, “XANX,” “LPDAX,” and “(E)RSGF/W” (Figure 1(b)), was found. These conserved motifs were shown to be critical to react with substrate AsA [42]. A neighbor-joining bootstrap tree was constructed based on the homologous APX proteins sequences from various plant species (Figure 1(a)). We revealed that they were divided into four groups and CaAPX was categorized into a cytosolic-type APX protein (Figure 1(a)).

### 3.2. Expression Pattern of* CaAPX* in* C. azalea*

We studied the expression patterns of* CaAPX* by qRT-PCR analysis in various tissues and different temperature stresses (Figure 1(c)). It was found that* CaAPX* was expressed in all tested tissues and the expression level of* CaAPX* in immature green fruit was the highest, with nearly 2.68 times higher than in mature leaf (Figure 1(c)). Notably, the expression levels in immature green fruit and mature leaf were higher than other tissue types (leaf bud, flower bud, flower, seed, and petal) (Figure 1(c)). To investigate the expression of* CaAPX* under temperature stresses, we examined the transcription levels in leaves after exposing to 4°C and 38°C, respectively, with 5 time points.

We showed that the expression levels of* CaAPX* in leaves were both upregulated under the low and high temperature treatment, reaching peaks at 8 h treatment. Interestingly, the expression levels both sharply declined in 12 h temperature stresses (Figures 1(d) and 1(e)).

### 3.3. Heterogeneous Expression of* CaAPX* Altered the Temperature Stress Tolerance and Displayed Elevated APX Activity and AsA Contents

**Figure 2:**
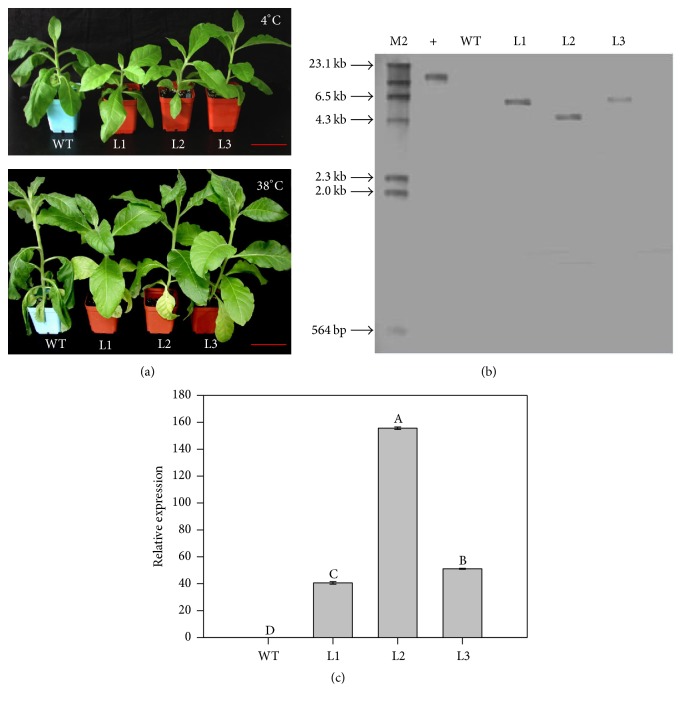
Analysis of transgenic plants. (a) Phenotypes of transgenic and WT plants under cold and hot stress treatments after 8 h. Upper panel, 4 degrees; bottom panel, 38 degrees. (b) Southern blot analysis of transgenic lines. (c) Detection of* CaAPX* transgenic tobacco by RT-PCR. Expression level of* CaAPX* gene in different lines (L1, L2, and L3) of transgenic and wild type (WT) plants. M2: *λ*-Hind III digested DNA marker; L1, L2, and L3: 3 lines of* CaAPX* transgenic tobacco; +: positive control of pBI121-CaAPX combined vector; −: water as the negative control; WT: wild type. The data are the means of four replicates and were compared by Duncan's multiple range tests.

To investigate the molecular functions of CaAPX, we generated transgenic tobacco plants with heterogeneous expression. We identified 20 positive transgenic lines by kanamycin selection and gene-specific amplification (Table S1, Figure S2). Three lines were randomly selected for southern blotting analysis, which all confirmed the single insertion of target construct (Figure 2(a)). We quantified the expression levels of* CaAPX* in these three transgenic lines using qRT-PCR. The results showed that expression level in L2 line was the highest, approximately 3.83 times higher than in L1 line, and L3 line had a higher expression level of around 1.23-fold compared to L1 line (Figure 2(b)). To study the tolerance to temperature stresses, transgenic plants were transferred into growth chamber at 4°C and 38°C, respectively. And phenotypes of treated plants were recorded every 15 min until significant wilting was emerged. After 8 h treatment under both 4°C and 38°C, the WT plants displayed obvious wilting symptoms, while transgenic lines displayed significantly enhanced performance (Figure 2(c)).

**Figure 3:**
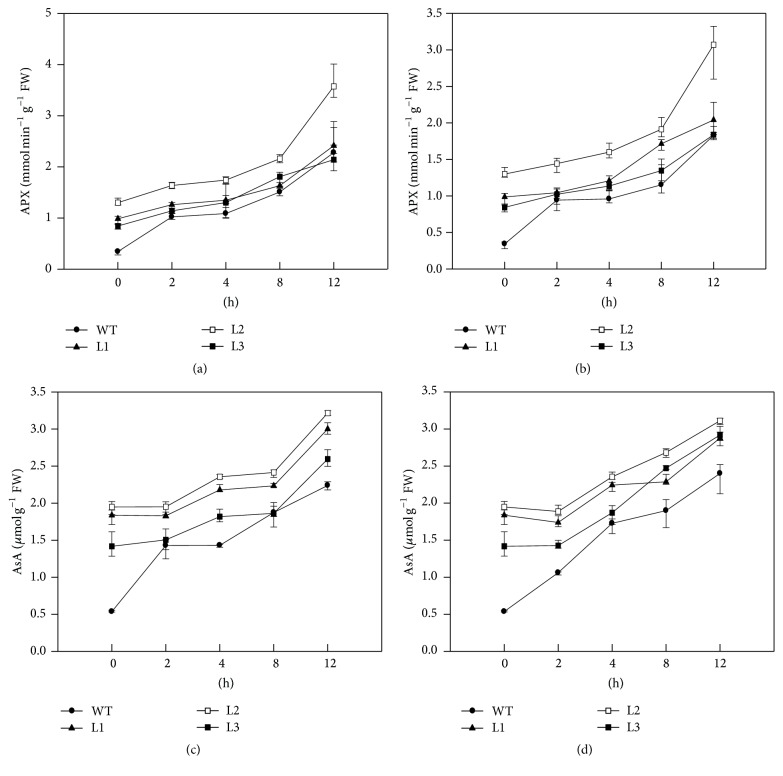
AsA content and APX activity in plants under temperature stress. (a) APX activities of transgenic and WT plants under 4°C temperature stress. (b) APX activities of transgenic and WT plants under 38°C temperature stress. (c) AsA contents of transgenic and WT plants under 4°C temperature stress. (d) AsA contents of transgenic and WT plants under 38°C temperature stress. L1, L2, and L3: 3 lines of* CaAPX* transgenic tobacco. WT: wild type. FW: fresh weight.

The activity of APX at different temperatures was examined in transgenic and WT plants. It was found that APX activity was much higher in three transgenic lines than in untreated WT plants (at 0 h in Figures 3(a) and 3(b)). The APX activities showed a similar increasing pattern in all tested transgenic lines as well as WT plants, upon cold and hot treatments (Figures 3(a) and 3(b)); meanwhile the L2 line displayed higher activity than the rest of assayed plants at each time point (Figures 3(a) and 3(b)). We further measured the contents of AsA in WT and transgenic plants under treatments. The results showed that, before treatments (at 0 h), all transgenic lines had significantly higher levels of AsA than WT (Figures 3(c) and 3(d)). Upon both 38°C and 4°C treatments, the AsA levels were increasing along with treatment durations in all transgenic plants and WT (Figures 3(c) and 3(d)). Also the L2 line had the highest AsA levels than other assayed plants at each time point (Figures 3(c) and 3(d)). Their results indicated that ectopic expression of* CaAPX* caused increased APX activity and AsA levels and enhanced the plant tolerances to cold and hot stresses. Line L2 displayed highest APX activity and AsA content, which is consistent with the accumulation patterns of* CaAPX* transcripts in transgenic plants (Figure 2(c)).

### 3.4. Overexpression of* CaAPX* Triggered Upregulation of Antioxidant Enzyme Activities

**Figure 4:**
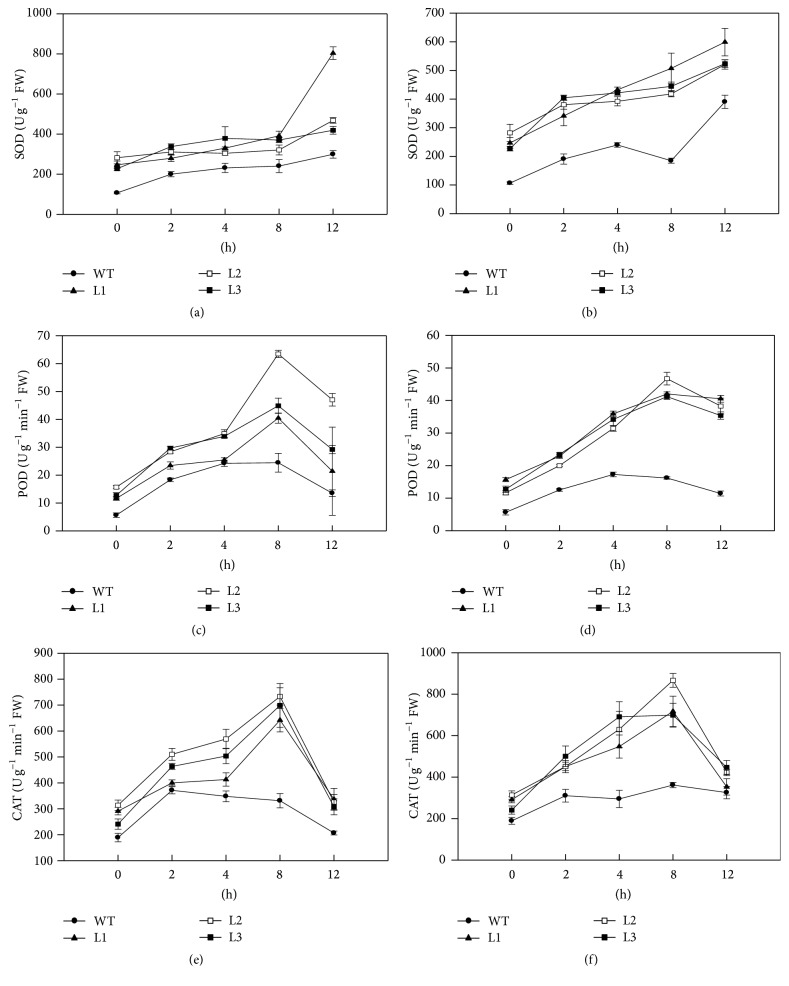
Activities of antioxidant enzyme in plants under temperature stress. (a) SOD activities of transgenic and WT plants under 4°C temperature stress. (b) SOD activities of transgenic and WT plants under 38°C temperature stress. (c) POD activities of transgenic and WT plants under 4°C temperature stress. (d) POD activities of transgenic and WT plants under 38°C temperature stress. (e) CAT activities of transgenic and WT plants under 4°C temperature stress. (f) CAT activities of transgenic and WT plants under 38°C temperature stress. L1, L2, and L3: 3 lines of* CaAPX* transgenic tobacco. WT: wild type. FW: fresh weight.

To study how the temperature stresses and* CaAPX* expression affected the ROS removing enzymes, the activities of SOD, POD, and CAT were measured. We showed that the SOD activities were upregulated before treatments (at 0 h in Figures 4(a) and 4(b)) in transgenic lines; there was a gradually increasing trend of SOD activity for each assayed plant during 38°C and 4°C treatments (Figures 4(a) and 4(b)); transgenic lines had higher SOD activities than WT at each timepoint of treatments (Figures 4(a) and 4(b)). The POD activities were upregulated in transgenic lines comparing to WT before treatments (at 0 h in Figures 4(c) and 4(d)), while the patterns of POD activities during treatments were different from SOD, which reached peaks at 8 h and declined at 12 h in transgenic plants (Figures 4(c) and 4(d)); in WT, the accumulation of POD activities was gradually increasing during 8 hours of treatments and decreased at 12 h (Figures 4(c) and 4(d)). The results of CAT analysis were similar to POD activities, except that CAT activities reached peaks at 8 h in all transgenic lines under both 38°C and 4°C treatments, and there was a sudden drop of activities at 12 h (Figures 4(e) and 4(f)). The activities of CAT in WT did not show strong induction under temperature treatments (Figures 4(e) and 4(f)).

### 3.5. Overexpression of* CaAPX* Reduced Accumulation of MDA, H_2_O_2_, and Electrolyte Leakage under Stresses

**Figure 5:**
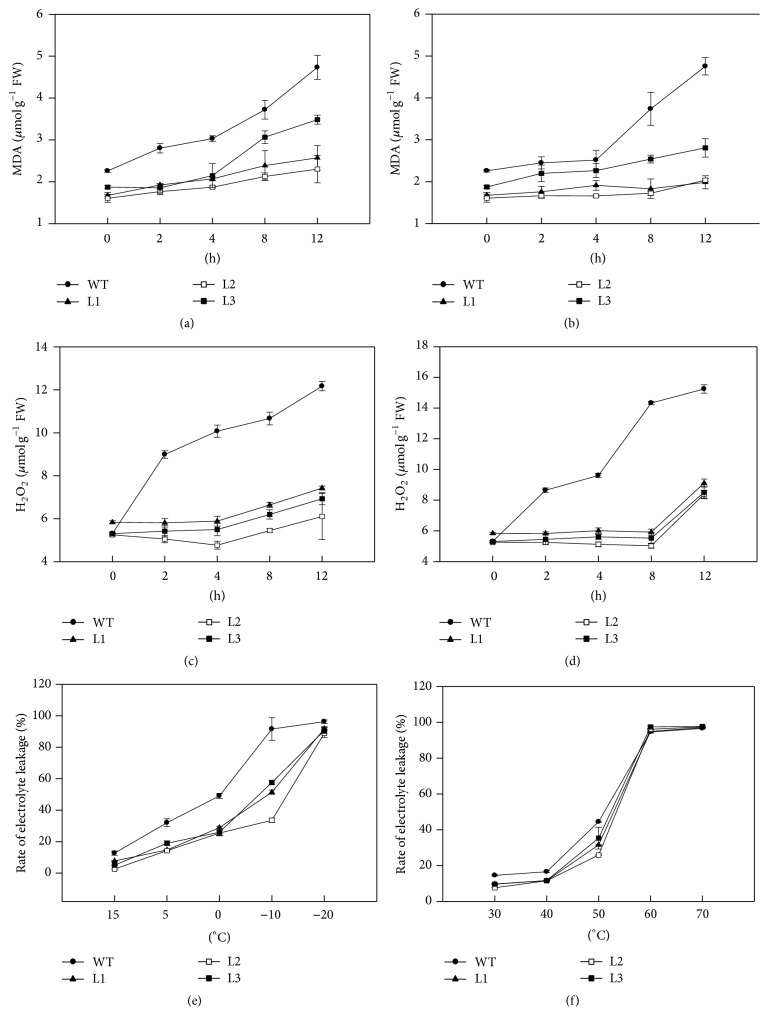
The contents of MDA, H_2_O_2_, and electrolyte leakage rates in plants under temperature stress. (a) MDA concentration of transgenic and WT plants under 4°C temperature stress. (b) MDA concentration of transgenic and WT plants under 38°C temperature stress. (c) H_2_O_2_ concentration of transgenic and WT plants under 4°C temperature stress. (d) H_2_O_2_ concentration of transgenic and WT plants under 38°C temperature stress. (e) Electrolyte leakage rates of transgenic and WT plants under low temperature stress. (f) Electrolyte leakage rat of transgenic and WT plants under high temperature stress. L1, L2, and L3: 3 lines of* CaAPX* transgenic tobacco. WT: wild type. FW: fresh weight.

To investigate the effects of altered ROS-scavenging activities (APX, CAT, and SOD) in transgenic plants, we measured the contents of MDA and H_2_O_2_ and the rate of electrolyte leakage. We found that MDA levels in WT plants were immediately induced when exposed to the abnormal temperatures (4°C and 38°C) and accumulated more dramatically compared with transgenic lines. The MDA contents in transgenic lines were significantly decreased, accounting for 71.09% to 82.91% in comparison to WT plants (0 h at Figures 5(a) and 5(b)).

Although there was no significant difference in H_2_O_2_ concentration between WT and transgenic plants incubated at room temperature (25°C), H_2_O_2_ concentration in WT plants increased markedly under 4°C and 38°C temperature stresses (Figures 5(c) and 5(d)). In transgenic plants, the H_2_O_2_ contents kept at the baseline level after 8 h treatments of temperature stresses (Figures 5(c) and 5(d)); but an increase at 12 h under 38°C was observed (Figure 5(d)). In addition, the rate of electrolyte leakage was higher in WT plants than in transgenic lines under the temperature ranges from 15°C to –20°C (Figure 5(e)). There was a slight rise in electrolyte leakage in all plants between 30 and 40°C (Figure 5(f)), and the conductivity rate increased dramatically to the highest level at 60°C and 70°C (Figure 5(f)), which suggested that the cells death of leaves had occurred since 60°C.

### 3.6. Parameters Associated with Chlorophyll Fluorescence in Leaves under Temperature Stresses

**Figure 6:**
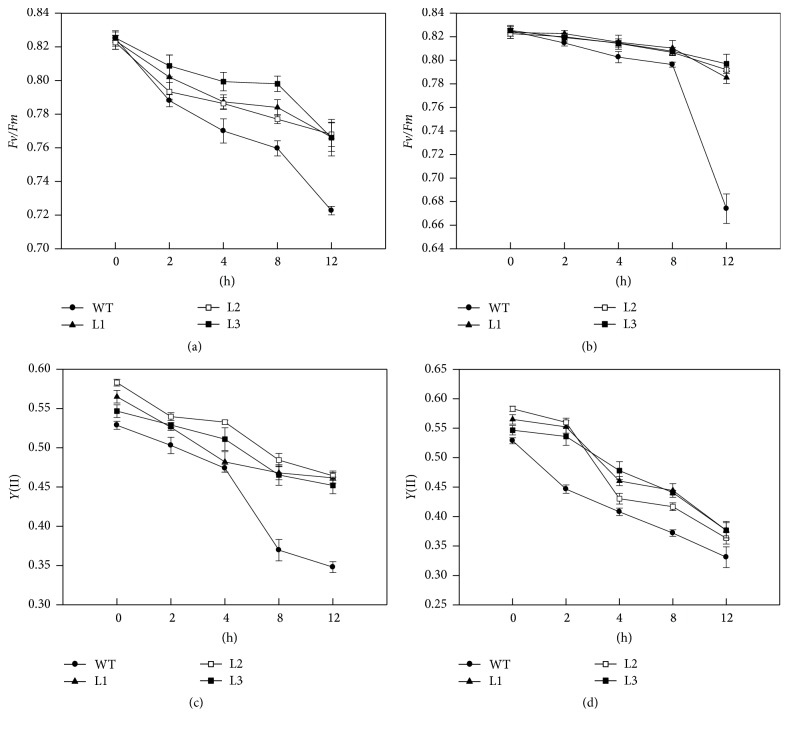
Photosynthetic parameters in plants under temperature stress. (a) *Fv*/*Fm* of transgenic and WT plants under 4°C temperature stress. (b) *Fv*/*Fm* of transgenic and WT plants under 38°C temperature stress. (c) *Y*(II) of transgenic and WT plants under 4°C temperature stress. (d) *Y*(II) of transgenic and WT plants under 38°C temperature stress. L1, L2, and L3: 3 lines of* CaAPX* transgenic tobacco. WT: wild type.

The photosynthetic electron transport (PET) chain is a sensitive process associated with cellular ROS homeostasis. To understand the regulatory roles of CaAPX under stresses, we measured several parameters such as *Fv*/*Fm* and *Y*(II) associated with chlorophyll fluorescence. Before the stress treatments (0 h at Figures 6(a) and 6(b)), the *Fv*/*Fm* was nearly the same between WT and transgenic plants. Upon temperature stresses, *Fv*/*Fm* decreased markedly under both 4°C and 38°C temperature stresses; the decline in WT plants was much accentuated (Figures 6(a) and 6(b)). The *Y*(II) was slightly higher in the transgenic lines compared to WT plants under control conditions, and the trend for *Y*(II) was similar to *Fv*/*Fm* under temperature stresses (Figures 6(c) and 6(d)). After 12 h treatment, the figures for transgenic lines were approximately 1.32- and 1.12-fold for average in comparison to WT sampling, respectively, at 4 and 38°C. Therefore, it was suggested that low and high temperatures significantly descended the parameters associated with chlorophyll fluorescence in both genotypes, in which the effects of stresses were more noticeable on WT plants than on the transgenic lines.

### 3.7. Divergent Induction Patterns of ROS-Scavenging Genes upon Cold and Heat Stresses

**Figure 7:**
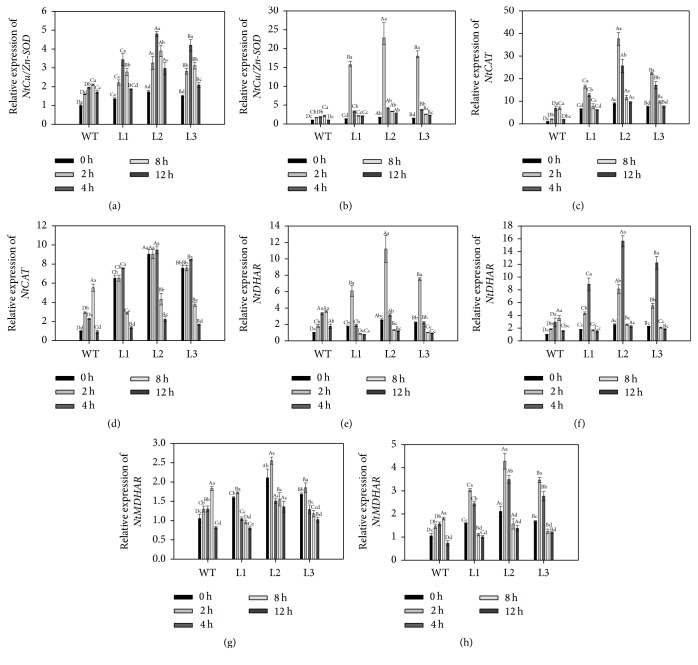
Expressions of* CaAPX* and defense-related genes in plants exposed to temperature stress. (a) Expression level of* NtCu/Zn-SOD* in transgenic and WT plants under 4°C temperature stress. (b) Expression level of* NtCu/Zn-SOD* in transgenic and WT plants under 38°C temperature stress. (c) Expression level of* NtCAT* in transgenic and WT plants under 4°C temperature stress. (d) Expression level of* NtCAT* in transgenic and WT plants under 38°C temperature stress. (e) Expression level of* NtDHAR* in transgenic and WT plants under 4°C temperature stress. (f) Expression level of* NtDHAR* in transgenic and WT plants under 38°C temperature stress. (g) Expression level of* NtMDHAR* in transgenic and WT plants under 4°C temperature stress. (h) Expression level of* NtMDHAR* in transgenic and WT plants under 38°C temperature stress. L1, L2, and L3: 3 lines of* CaAPX* transgenic tobacco; WT: wild type. Different lowercase letters represent significant differences among the* CaAPX* gene transgenic and WT plants, whereas different capital letters represent significant differences among the stress treatments, at a confidence level of 0.05. The data are the means of four replicates and were compared by Duncan's multiple range tests.

The elevated levels of ROS by temperature stresses are important signals to control the expression of ROS-scavenging enzymes [2, 5, 43]. To understand the ROS signal transduction in transgenic* CaAPX* plants under stresses, we analyzed the transcripts levels of tobacco ROS-scavenging genes,* NtCu/Zn-SOD*,* NtCAT*,* NtDHAR*, and* NtMDHAR*, in response to temperature stresses. We showed that, before the temperature stress treatments, the expression of* NtCAT* was obviously upregulated in transgenic lines compared with WT plants (Figure 7(c)), while expression of* NtCu/Zn-SOD*,* NtDHAR*, and* NtMDHAR* was not significantly induced in* CaAPX* transgene plants. Upon cold and hot stresses treatments, the expression levels of all assayed genes were upregulated at some time points, but the patterns of induction between cold and hot stresses were mostly different (Figures 7(a)–7(h)). For instance, remarkable induction of* NtCu/Zn-SOD* was detected only at 2 h treatment of 38°C and expression dropped thereafter in* CaAPX* transgenic plants; under 4°C treatments moderate inductions were observed in both WT and transgenic plants (Figures 7(a) and 7(b)). In WT, the expression of* NtCAT* was induced significantly after 2 h of hot treatment (Figure 7(d)), while, in* CaAPX* transgenic plants, the expression of* NtCAT* did not change after 2 h and 4 h and greatly reduced at 8 h and 12 h of hot treatment. Under cold treatment, the expression of* NtCAT* was upregulated in both WT and transgenic plants (Figure 7(c)). These results suggested the* CaAPX* overexpression affected the ROS mediated regulation of SOD and CAT expression, and the hot and cold stresses had different responsive mechanisms of signaling. Meanwhile, the expression levels of* NtDHAR* and* NtMDHAR* displayed similar induction patterns between WT and transgenic plants under both cold and hot stresses (Figures 7(e)–7(h)), suggesting a different regulatory pathway was involved.

### 3.8. *CaAPX* Overexpression Plants Displayed Greater Thermotolerance in Seed Germination Assay

**Figure 8:**
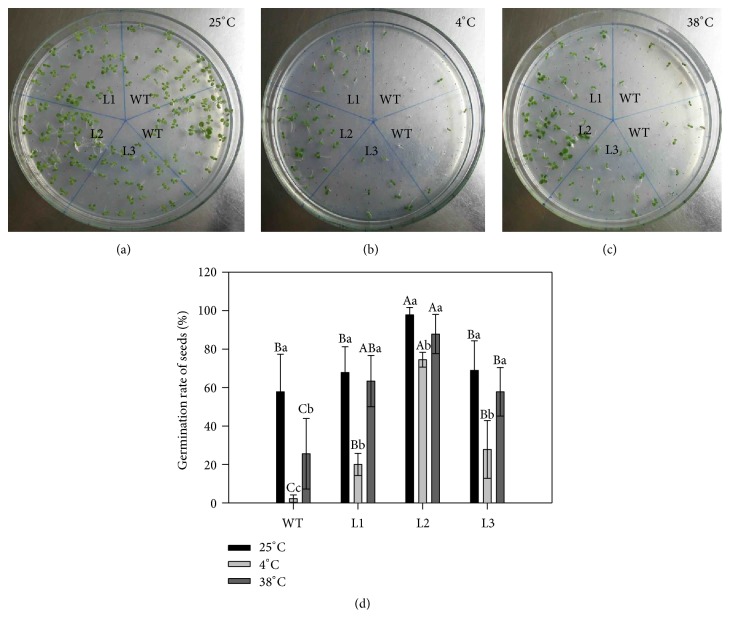
Germination rates of T1 generation seeds under temperature stress. (a) The state of seeds germination under 25°C. (b) The state of seeds germination under 4°C. (c) The state of seeds germination under 38°C. (d) Rate of seeds germination of T1 generation transgenic and WT seeds under different temperature treatments. L1, L2, and L3: 3 lines of* CaAPX* transgenic tobacco; WT: wild type. Different lowercase letters represent significant differences among the* CaAPX* transgenic and WT plants, whereas different capital letters represent significant differences among the stress treatments, at a confidence level of 0.05. The data are the means of four replicates and were compared by Duncan's multiple range tests.

To further investigate the thermotolerance effects of* CaAPX* in transgenic plants, we tested the germination of transgenic lines. The assayed lines were confirmed by T2 segregation analysis through PCR amplification of progenies (Figure S3). No segregation was found for all three lines. Then we tested the germination rates of seeds on the MS medium containing antibiotics selection at 25°C, 4°C, and 38°C, respectively. We found that the germination rates of transgenic lines were a little higher than from WT seeds at 25°C treatment but not significant (Figures 8(a), 8(b), and 8(c)).

Under 4°C and 38°C temperature stresses, the rates of transgenic lines were significantly higher than WT seeds (Figures 8(b), 8(d) and 8(c)). Particularly, under 4°C, almost no germination was found for WT plants, but the strong line (L2) showed a comparable rate to WT under normal condition (Figure 8(d)). The results indicated that overexpressing* CaAPX* significantly enhanced temperature stresses tolerance and improved the seed germination rate in transgenic lines under temperature stresses.

## 4. Discussions

Temperature stress, namely, low and high temperatures, often caused negative impacts on plant metabolism, cellular homeostasis, and major physiological processes [2]. Previous studies demonstrated that ROS is one of the main factors causing oxidative damage to plants exposed to environmental stresses [29]. Antioxidant enzymes play a significant role in eliminating toxic levels of ROS, generated during stress from living cells [44]. In stressful conditions, plants generally increased the APX activity and the elevated activity level usually correlates with increased stress tolerance [3]. During high and low temperature treatments, many physiological changes occur that result in increased expression of antioxidant enzyme genes to protect from oxidative stress. APX utilizes AsA and its specific electron to reduce H_2_O_2_ to H_2_O, and overexpressing APX might play a critical role in growth and when subjected to high and low temperature. Previous research indicated that APX was highly responsive to temperature stresses and played an important role in the scavenging of ROS in plants. For example, the* APX1* expression of* A. thaliana* significantly increased after being treated with heat stress [45]; Padaria et al. [46] found that* TapAPX* of a thermotolerant wheat cv. Raj3765 displayed up to 203-fold level of expression at 42°C heat stress exposure. The expression level of* tAPX* during cold acclimation of wheat was upregulated [47]; the expression of* A. andraeanum APX* mRNA was evidently elevated by cold stress [48];* OsAPX2* expression of* Oryza sativa* was also developmental- and spatial-regulated and was induced by cold stress [49]. In this study, the expression levels of* CaAPX* were both remarkably upregulated under low and high temperature treatment (Figures 1(d) and 1(e)). It demonstrated that* CaAPX* was induced by heat and cold. We found that overexpression of* CaAPX* increased tolerance to temperature stresses, and transgenic tobacco grew better than wild type under 4°C and 38°C temperature stresses (Figure 2(a)). We showed that the activity of APX and the content of AsA were indeed remarkably enhanced in transgenic plants than those in wild type plants (Figures 3(a)–3(d)), suggesting their roles in scavenging H_2_O_2_ (Figures 5(c) and 5(d)).

Previous studies indicated that antioxidant enzyme activities were tightly associated with the expression of antioxidant enzyme genes, which could be upregulated after the plants were treated with temperature stresses. For instance, Lin et al. [50] reported that* CAT* and* APX* expression profiles were well matched with the data for CAT and APX enzyme activities in the broccoli and Chinese cabbage plants, respectively. And overexpressing chloroplast* APX* in cotton has conferred higher photochemical activity of photosystem II (PSII) and stronger antioxidant capacity to transgenic plants [51]. Overexpression of* CaAPX* also enhanced the activities of SOD, POD, and CAT in transgenic tobacco plants than that in the wild type plants under 4°C and 38°C temperature stresses (Figures 4(a)–4(f)). Furthermore, it was demonstrated that the high activities of SOD, POD, and CAT could result in producing the high level of AsA, which could alleviate photoinhibition to protect the plants [52]. Moreover, the high level of AsA could detoxify ROS effectively, further reduce the damage to membrane lipids, enhance the repair of PSII, and protect photosystem. Zhou et al. [53] concluded that *Fv*/*Fm* is an early indicator of temperature stress tolerance in tomato and that this provides effective and reliable information about leaf photosynthetic performance. Hence, several parameters such as *Fv*/*Fm* (Figures 6(a) and 6(b)) and *Y*(II) (Figures 6(c) and 6(d)) associated with chlorophyll fluorescence in this study were decreased under extreme temperature stresses, but these effects were more accentuated in wild type plants. These results manifested that* CaAPX* is a key component to balance the cellular ROS homeostasis and could be an ideal target for improving tolerance to temperature stresses.

The transcript levels of* NtCu/Zn-SOD*,* NtCAT*,* NtDHAR*, and* NtMDHAR* genes significantly increased on exposure to stress in transgenic tobacco plants (Figure 7). The critical role of APX in stress tolerance and upregulation of* Cu/Zn-SOD*,* CAT*,* DHAR*, and* MDHAR* transcripts have been reported [54] under stress. Variations in physiological parameters potentially contribute to the complex phenotype of cold and heat tolerance; however, a clear relation between stress and coexpression of antioxidant genes has so far not been evident. In this study, the expression pattern of* NtCu/Zn-SOD* and* NtCAT* in times was specific to two genotypes and showed no direct correlation with the corresponding enzymes activity. This result was likely because of the complex regulation mechanisms of gene expression; gene expression cannot be directly correlated with enzyme activity [55].

*CaAPX* was expressed in all tested tissues (leaf, leaf bud, flower, flower bud, petal, immature green fruit, and seed embryo) but was much more abundant in the immature green fruit relative to other tissues (Figure 1(c)). Wang et al. [56] showed that a wide range of* MaAPX1* transcript expression levels among the different tissues in banana, with relatively strong expression, was observed in leaves and roots. Our results indicated that* CaAPX* might have different functions in different tissues during the growth and development of* C. azalea*. To test the thermotolerance and cold-tolerance of transgenic plants, we employed a seed germination assay. Sun et al. [57] reported that overexpression of* StAPX* improved seed germination, and Faize et al. [58] showed that overexpression of* cytapx* also promoted seed germination and germination rates. In our conditions, we found that overexpressing* CaAPX* significantly enhanced temperature stresses tolerance (Figures 8(a), 8(b), and 8(c)) and improved the seed germination rate in transgenic plants (Figure 8(d)) under hot and cold stresses, which was consistent with the other previous plants. Overall, this work characterized the functions of CaAPX in response to heat and cold stresses. The results provided comprehensive information about the orchestration of cell ROS homeostasis mediated by CaAPX, which could give some insights into rational engineering of plants toward temperature tolerances.

## Supplementary Material

The supplementary information includes 1 table and 2 figures. Table S1, Primers used in this study. Figure S1, Nucleotide sequence of CaAPX and its postulated amino acid sequence. Figure S2. Detection of CaAPX transgenic tobacco by RT-PCR. Figure S3. Detection of genetic stability of T1 generation plants by PCR analysis.

## Additional Points

*Key Message*. CaAPX encodes a cytosolic-type APX. Ectopic expression of CaAPX enhanced heat and cold tolerances and displayed elevated ROS-scavenging activities. CaAPX is central to ROS homeostasis and temperature tolerance.
