# Supplementary material for: Overexpression of CaAPX Induces Orchestrated Reactive Oxygen Scavenging and Enhances Cold and Heat Tolerances in Tobacco
Source: Biomed Res Int. 2017 Mar 13;2017:4049534. doi: 10.1155/2017/4049534 (PMC5366785; doi:10.1155/2017/4049534)
Supplement: Supplementary file 1 — The supplementary information includes 1 table and 2 figures. Table S1, Primers used in this study. Figure S1, Nucleotide sequence of CaAPX and its postulated amino acid sequence. Figure S2. Detection of CaAPX transgenic tobacco by RT-PCR. Figure S3. Detection of genetic stability of T1 generation plants by PCR analysis. [file 4049534.f1.docx]

**Table S1**

Primers used in this study

| Primer ID | sequence（5’-3’） | Usage |
| --- | --- | --- |
| P1 | GCATGGCACTCTGCTGGTAC | Conservative region for initial cloning |
| P2 | CATCCGCAGCATATTTCTCAAC |  |
| P3 | GTTCAGAGAGCTTCATGTG | 3’RACE-Outer |
| P4 | CATCCGCAGCATATTTCTCA AC | 3’RACE-Inner |
| P5 | GCATGGCACTCTGCTGGTAC | 5’RACE-Outer |
| P6 | ACTGGAGGACCTGATGTTCC | 5’RACE-Inner |
| P7 | CTACTTCACGGAACTCCTGAC | Quantitative Real-time PCR of *CaAPX* in *C. azalea* |
| P8 | GAATGCATCCTCATCCGCAGCAT |  |
| P9 | GACTCAACACGGGGAAACTTACC | Camellia reference gene |
| P10 | CAGACAAATCGCTCCACCAAC |  |
| P11 | GCTCTAGAATGGGGAAGTGCTATC  *Xba* I | Plant expression vector construction; PCR identification of sense *CaAPX* transgenic tobacco |
| P12 | GCGGATCCTTAGGCTTCAGCAAAC  *BamH* I |  |
| P13 | ACTGGAGGACCTGATGTTCC | Gene-specific probes for Southern blotting |
| P14 | GAATGCATCCTCATCCGCAGCAT |  |
| *NtCu/Zn-SOD-*F | CTGGTGATCTTGGTAACATCACA | Quantitative Real-time PCR of *NtCu/Zn-SOD* in transgenic tobacco and WT plants |
| *NtCu/Zn-SOD-*R | CCAAGATCATCAGGATCAGCGT |  |
| *NtCAT-*F | CCTATGTGAAGTTCCACTGGAAGC | Quantitative Real-time PCR of *NtCAT* in transgenic tobacco and WT plants |
| *NtCAT-*R | CGGCAATAGAGTCATAGAGGTCT |  |
| *NtDHAR-*F | TCAAGGCTCACGGACCATATGT | Quantitative Real-time PCR of *NtDHAR* in transgenic tobacco and WT plants |
| *NtDHAR-*R | GCACATGACTCAAGCTTTCAGG |  |
| *NtMDHAR-*F | GGATTGACTCTGGTAAGCTGA | Quantitative Real-time PCR of *NtMDHAR* in transgenic tobacco and WT plants |
| *NtMDHAR-*R | AATGCCTCTTCCACTGAGGATG |  |
| *NtEF1a*-F | CGGTTAAGGATCTCAAGCGT | Tobacco reference gene |
| *NtEF1a*-R | GAACTGGAGCATATCCATTGCC |  |


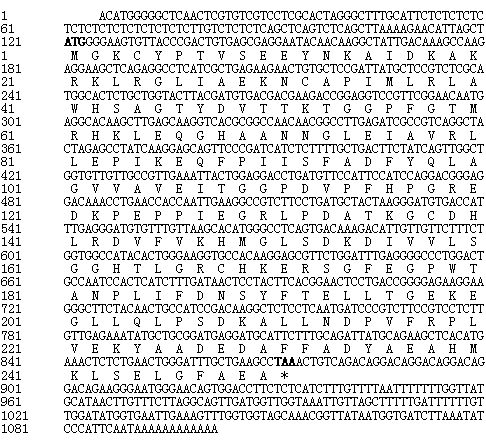


**Figure S1.** Nucleotide sequence of *CaAPX* and its postulated amino acid sequence. ATG, the initiation codon; TGA, the terminator codon.

100bp→

500bp→

1000bp→

2000bp→

750bp→

200bp→


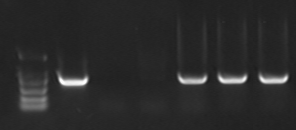


M1 + － WT L1 L2 L3

**Figure S2.** Detection of *CaAPX* transgenic tobacco by RT-PCR. M1: DL2000 DNA Marker; L1, L2, L3: 3 lines of *CaAPX* transgenic tobacco; +: Positive control of pBI121-*CaAPX* combined vector; -: ddH_2_O; WT: Wild type.

M1 + －WT 1 2 3 4 5 6 7 8 9 10 11 12 13 14 15 16 17 18 19 20


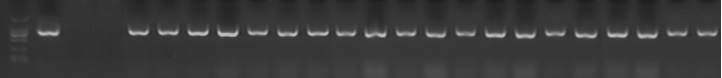

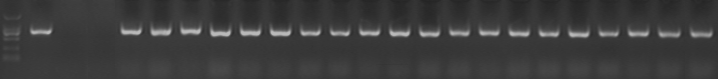

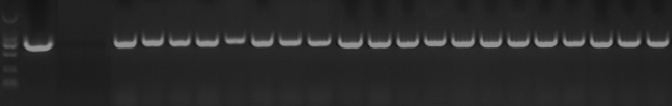


L1

L2

L3

100bp→

750bp→

100bp→

2000bp→

2000bp→

100bp→

2000bp→

250bp→

750bp→

750bp→

250bp→

250bp→

**Figure S3.** Detection of genetic stability of T1 generation plants by PCR analysis. M1: DL2000 DNA Marker; 1~20: 20 *CaAPX* gene transgenic tobacco plants; +: Positive control of pBI121-*CaAPX* combined vector; -: ddH_2_O; L1, L2, L3: 3 lines of *CaAPX* transgenic tobacco; WT: Wild type.
